# Measuring flexibility: A text-mining approach

**DOI:** 10.3389/fpsyg.2022.1093343

**Published:** 2023-01-18

**Authors:** Katalin Grajzel, Selcuk Acar, Denis Dumas, Peter Organisciak, Kelly Berthiaume

**Affiliations:** ^1^University of Denver, Denver, CO, United States; ^2^University of North Texas, Denton, TX, United States; ^3^University of Georgia, Athens, GA, United States

**Keywords:** divergent thinking, flexibility, text-mining, psychometrics, creativity

## Abstract

In creativity research, ideational flexibility, the ability to generate ideas by shifting between concepts, has long been the focus of investigation. However, psychometric work to develop measurement procedures for flexibility has generally lagged behind other creativity-relevant constructs such as fluency and originality. Here, we build from extant research to theoretically posit, and then empirically validate, a text-mining based method for measuring flexibility in verbal divergent thinking (DT) responses. The empirical validation of this method is accomplished in two studies. In the first study, we use the verbal form of the Torrance Test of Creative Thinking (TTCT) to demonstrate that our novel flexibility scoring method strongly and positively correlates with traditionally used TTCT flexibility scores. In the second study, we conduct a confirmatory factor analysis using the Alternate Uses Task to show reliability and construct validity of our text-mining based flexibility scoring. In addition, we also examine the relationship between personality facets and flexibility of ideas to provide criterion validity of our scoring methodology. Given the psychometric evidence presented here and the practicality of automated scores, we recommend adopting this new method which provides a less labor-intensive and less costly objective measurement of flexibility.

## Introduction

For the last century, psychologists have emphasized the importance of cognitive flexibility in creative thinking. Flexibility of thought is a metacognitive process related to shifting in thinking and alternating between controlled and spontaneous cognitive processes (Yu et al., 2019; Preiss, 2022). Shifting between different processes is central to creative idea generation and has been compared to mindful mind wandering (Martindale, 1999; Preiss and Cosmelli, 2017; Murray et al., 2021). Flexibility does not just provide plasticity of cognition to allow for creative performance, but it also supports the consolidation of knowledge which affords one to achieve the highest potential of learning (Kroes and Fernández, 2012; Beckmann, 2014).

Creativity studies have provided empirical links between individuals’ creative outcomes and the flexibility of their thinking (Zabelina and Robinson, 2010; De Dreu et al., 2011; Kenett and Faust, 2019) showing that individuals who are capable of thinking in a flexible way may be more likely to produce highly creative ideas and products (Hass, 2017; Acar et al., 2019; Kenett and Faust, 2019; Zhang et al., 2020). Indeed, genuine scientific and artistic achievement has been associated with flexibility of cognition (Zabelina and Robinson, 2010; Zabelina et al., 2019; Griffith, 2022) and flexibility of the lexical network (Kenett et al., 2016, 2018; Cosgrove et al., 2021). Additionally, flexibility has also been used to differentiate between gifted and non-gifted children in performance areas such as writing, crafts, art, and public presentation (Shore, 2000; Keleş and Yazgan, 2022).

Research on flexibility also supports fundamental cognitive and developmental inferences, including the idea that exposure to novel and unexpected experiences through diverse life events may increase cognitive switching (Ritter et al., 2012; Chen et al., 2022). Indeed, openness to experiences has been found to be a good predictor of creativity (Feist, 1998; Abraham, 2018; Runco and Pritzker, 2020). This personality characteristic is a domain of the five-factor model of personality (McCrae and John, 1992; Goldberg, 1993; John et al., 2008) and is defined as curiosity, imaginativeness, creativity, trying new things, and unconventional thinking (John and Srivastava, 1999). The five factors of the model include openness (to experiences), conscientiousness, extraversion, agreeableness, and neuroticism. Costa and McCrae (1995) found that openness is positively associated with creativity while conscientiousness is negatively associated. Feist (1998) also found a similar effect using a meta-analysis of studies focusing on artists and scientists. Several other studies have confirmed the relationship between openness and extraversion with divergent thinking performance (Carson et al., 2003; Dumas et al., 2020a; Asquith et al., 2022).

### Assessing flexibility of idea generation

Divergent thinking tasks have been widely used to evaluate domain general creative ability (Plucker et al., 2004; Silvia et al., 2008; Karwowski et al., 2021). In DT tasks, a prompt or a specific problem is presented requiring ideas to be generated by way of exploring many possible solutions (Runco and Acar, 2012; Reiter-Palmon et al., 2019; Kanlı, 2020). Verbal DT tasks contain open-ended prompts such as unusual uses for common objects (e.g., brick, bottle, table), instances or examples of common descriptors (e.g., loud, bright, and cold), similarities between common concepts (e.g., milk and meat), and consequences of imaginary events (e.g., clouds had strings).

The DT idea generation process is thought to be free-flowing, non-linear where many possible solutions are explored in a random manner (Kenett et al., 2018; Gray et al., 2019). Because DT often leads to solutions that are unique and original DT has been associated with creativity (Guilford, 1962; Acar et al., 2019; Peterson and Pattie, 2022). DT is an estimator of creative potential and not a measure of creativity (Runco, 1993; Runco, 2010; Carruthers and MacLean, 2019). It reflects the process of idea generation and its product in an easily measurable format (Milgram, 1991; Runco, 1993; Acar and Runco, 2012; Reiter-Palmon et al., 2019).

Flexibility is a dimension of DT with high theoretical importance, although it is only measured in a minority of published work in the creativity research literature. Flexibility is generally conceptualized in two major ways in contemporary literature (Acar and Runco, 2017). The more traditional and historical definition addresses the grouping of products into categories (Guilford, 1968; Mastria et al., 2021). In this context, flexibility is operationalized as the number of unique categories to which ideas belong (Guilford, 1975; Runco, 1986a; Runco and Okuda, 1991; Runco, 2013; Johnson et al., 2021). An alternate view of flexibility has focused on the shifting or switching in the process of ideation (Torrance, 1974; Yu et al., 2019; Preiss, 2022).

**Figure 1:**
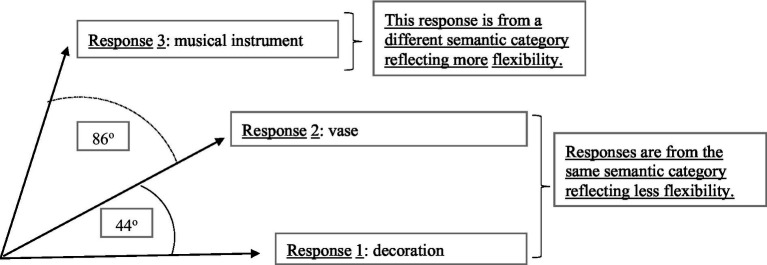
Measuring flexibility as a distance between consecutive responses. These responses are alternate uses of a bottle scored using GLoVe text-mining system.

The importance of flexibility is in its ability to differentiate the creative quality of participant responses above and beyond originality (Nijstad et al., 2010; Kenett et al., 2018; Forthmann et al., 2019; Reiter-Palmon et al., 2019). For instance, when asking participants to create unusual uses to the prompt of “bottle,” the answers of “decoration” and “vase” would be considered to be in the same semantic category as they are using the bottle in somewhat similar ways. Therefore, each individual idea might be considered to have a certain degree of originality, but those two responses together would not exhibit high flexibility. However, the answer of “musical instrument” to the same prompt (i.e., bottle) is in a different semantic category as it relates to a different use all together, therefore resulting in a higher flexibility score (see Figure 1). The quantification of flexibility, therefore, is a methodological choice that can account for the close relations between responses in the same cluster and ensures that participants who generate ideas that are truly divergent from one another are captured in the scoring method (Acar and Runco, 2019).

### Scoring idea generation

Together, originality and flexibility quantify the creative quality of participant responses to a DT task. As such, their scoring has historically been less objective and more time consuming compared to the scoring of fluency, which refers to more quantitative aspects of idea generation (i.e., the total count of ideas). Several scoring methods exist for flexibility, and each of those scoring methods presents unique advantages and weaknesses (Silvia et al., 2008; Barbot et al., 2019; Reiter-Palmon et al., 2019). Uniqueness scoring is one of the most widely used methods to evaluate the quality of idea generation in DT tasks (Silvia et al., 2008). Using this approach, a point is given for the use of a word from a category that has not occurred anywhere else within the analytic sample (Acar and Runco, 2019). As this method relies on the characteristics of a given sample for its scoring, the results are necessarily highly sample-dependent (Forthmann et al., 2019).

Also commonly used in the extant literature (e.g., Silvia, 2011), subjective scoring overcomes the sample dependency issues of uniqueness scoring. This type of scoring relies on trained human judges to rate the uniqueness of an idea in relation to each other to assess flexibility. Subjective scoring eliminates the need for the construction of a reference sample as the comparison and instead, answers are scored in relation to the human rater’s own experience. This scoring method is dependent on the raters’ perception of creativity and can produce inconsistent and biased results (Silvia et al., 2009; Primi et al., 2019; Reiter-Palmon et al., 2019). Hass and Beaty (2018) found that variances in scores obtained from 80 raters differed based on the task, such as for AUT the variance in ratings was 27% while in the case of consequences was 60%, and items, 10% in the case of AUT. For this reason, multiple raters are always needed in his method in order to ascertain inter-rater reliability and to understand the way in which idiosyncrasies among the raters are influencing the scoring (Kaufman et al., 2008; Guo et al., 2019). Forthmann et al. (2017) examined rater agreement scoring DT tasks and found that rater disagreement was higher when “be creative” instructions were used compared to standard instructions. They also found that rater disagreement was higher for less complex compared to more complex ideas across the different instructions.

Determining the cluster size when scoring flexibility also presents an additional problem in this scoring method (Hass, 2017). As cluster sizes are set arbitrarily therefore different scores are obtained when clusters are set to be wide, such as including all animals, or narrow, such as only including mammals. The size of the category or class during flexibility scoring is directly related to another phenomenon observed in creativity scoring termed the fluency bias (Plucker et al., 2004; Benedek et al., 2012; Forthmann et al., 2020). It has been observed that participants who generate a greater number of ideas are more likely to receive a higher originality score in comparison to those who generate fewer ideas, in part because more original ideas tend to arise later in the ideation process (Beaty and Silvia, 2012; Gonçalves and Cash, 2021). Flexibility suffers from the same confound as fluency in which a greater number of ideas generated increases the likelihood of those ideas belonging to different conceptual or semantic categories. Although research on the phenomenon is sparse in the context of flexibility, Acar et al. (2022), in a recent meta-analytic review, found a strong correlation between fluency and flexibility (*r* = 0.79) and concluded that fluency bias in the context of flexibility is even more profound than in the case of originality. Several summarization methods may function as solutions to the quantity-quality confound, such as using average quality (i.e., mean originality or mean flexibility), or only counting the most creative idea a participant produces (i.e., maximum originality or maximum flexibility), have been suggested (Runco and Acar, 2012; Acar and Runco, 2019; Reiter-Palmon et al., 2019). Therefore, in this study, we employed both average and maximum scoring methods to account for the effect of fluency bias (Table 1 lists all scoring methods used in this study).

**Table 1:** The variety of scoring methods used in the study.

Scoring methods		Definition
Measure-based scoring	Text-mining (OCS)	Scores are calculated by measuring semantic distance between responses using the https://openscoring.du.edu/about site which is based on the GLoVe text-mining system.
	Publisher-generated (Pub)	Scores are assessed based on the frequency of responses and obtained from the publisher (STS) of the TTCT. Scores are calculated as the sum of all responses (e.g., sum).
Aggregation methods	Mean	Scores are calculated as the average of all scores across all responses given to an item or task. Utilized to minimize fluency confound when measuring originality and flexibility (Runco and Acar, 2012; Forthmann et al., 2019).
	Maximum	The highest scoring response is generated to an item or task. Utilized to minimize fluency confound when measuring originality and flexibility (Runco and Acar, 2012; Forthmann et al., 2019).
	Sum	The sum of the scores of all responses generated to each item or task. This method is traditionally applied when scoring DT measures (e.g., TTCT, publisher-generated).
Scored dimensions	Flexibility (Flex)	Operationalized as shifting or switching in the process of ideation (Torrance, 1974; Yu et al., 2019; Preiss, 2022) or the number of unique categories to which ideas belong (Guilford, 1975; Runco, 1986a; Runco and Okuda, 1991; Runco, 2013; Johnson et al., 2021).
	Originality (Orig)	It is the most often used dimension and it refers to the uniqueness or novelty of an idea (Dumas and Dunbar, 2014; Beketayev and Runco, 2016).
	Fluency (Flu)	It is defined as the number of responses calculated as the total count of ideas generated on each item or task.

Parenthesis denotes the abbreviation used throughout the manuscript.

### Text mining-based scoring for idea generation

In addition to the clear sample dependency issues, confounds with the quantity of ideas, and human rater-biases that have plagued the scoring of creative quality dimensions (i.e., originality and flexibility) in the past, these traditional scoring methods are also highly labor intensive and costly. As verbal DT measures are designed to collect textual answers to open-ended prompts, each answer must be carefully read and scored by multiple human raters (Silvia, 2011; Reiter-Palmon et al., 2019). However, recent advances in machine learning have enabled creativity researchers to use associative network-based and text-mining based methods to assess creative ideations. Semantic network (SN) based methods rely on machine learning analysis which estimates relationships among words or concepts determined by their association within natural language. Words with close semantic association form clusters or neighborhoods which in turn form association networks (Rapp and Samuel, 2002; Pranoto and Afrilita, 2019). Acar and Runco (2014) were the first to examine the application of semantic network based methods in creativity research using the alternative uses task (AUT). They found that individuals with higher creative attitudes generated responses forming a more remote cluster compared to less creative individuals. Beketayev and Runco (2016) were the first to examine flexibility using semantic network based machine learning methods. They found a strong correlation between the number of categories into which responses fell and traditional uniqueness flexibility scores. Kenett et al. (2018) examined semantic networks of high creative individuals which demonstrated stronger links, therefore exhibiting more flexibility compared to low creative individuals. Focused on cognitive aspects of aging, Cosgrove et al. (2021) found diminished flexibility of the semantic networks of older adults compared to younger adults. SN based methods are useful for visualization and exploration of the relationships between responses. However, it lacks the functional ability to produce scores for individual responses, therefore, limiting practical applications.

Text-mining based methods are capable of denoting similarities between words numerically. There are several open accesses, web-based tools available for text-mining scoring to researchers through the Pennsylvania State University (Beaty and Johnson, 2021; http://semdis.wlu.psu.edu) or through the University of Denver (Organisciak and Dumas, 2020; https://openscoring.du.edu). To emulate natural language, text-mining methods collect a large number of textural documents or corpora (Cai et al., 2018). Forster and Dunbar (2009) were the first researchers to apply this method to creativity measurement, specifically to score divergent thinking tasks. They found a significant correlation between human-rated originality scores and originality scores obtained through text-mining on the Alternative Uses Test (AUT). Soon after that first study, Heinen and Johnson (2018) found that text-mining based creativity scores significantly correlated with subjective ratings of participants’ answers on a 20 and a 60-prompt creativity test. Dumas and Dunbar (2014) were the first to assess the construct reliability of text-mining based originality scores, and later examined the reliability of text-mining based originality scores even when controlling for fluency. To emulate natural language, text-mining methods collect a large number of textural documents or corpora. The matrix consists of rows and columns representing each word and each document, respectively (Cai et al., 2018). Words are then weighted in the matrix based on co-occurrences such that words that occur less often have greater weight, or emphasis in the model, and words occurring more often (i.e., common words) have less. Responses are represented as the vector of their summed and weighted words in semantic space. Semantic similarity between terms can be calculated as the cosine of the angle between their vectors (Oniani, 2020; Dumas et al., 2020a).

### A proposed text-mining based measure of flexibility (OCS-flex)

Semantic distances measured between consecutive responses provide information about their semantic relationship. Small distances between words indicate semantic relatedness while large distances demonstrate unrelatedness expressions (Forthmann et al., 2019; Acar et al., 2021). Related words can be reasonably assumed to originate from the same semantic category while unrelated ideas are generated from different semantic categories, therefore, reflecting flexibility of thought (Beaty et al., 2021; Johnson et al., 2021).

Our proposed scoring method is based on this characteristic of flexibility, namely the relation between the semantic distance between the adjacent responses produced for the same prompt and the category to which these ideas belong. This relationship is numerically denoted in text-mining as the cosine of the angle between word vectors (Deerwester et al., 1990). For example, in Figure 1, the first response “decoration” given to the prompt “bottle” in an AUT exercise is in the same semantic category as the second response “vase.” This close semantic association is denoted by the cosine between the angles of the two responses (44^o^). However, the third response “musical instrument” is in a different semantic category from the first two, denoted by the larger cosine between their angles (86^o^).

**Figure 2:**
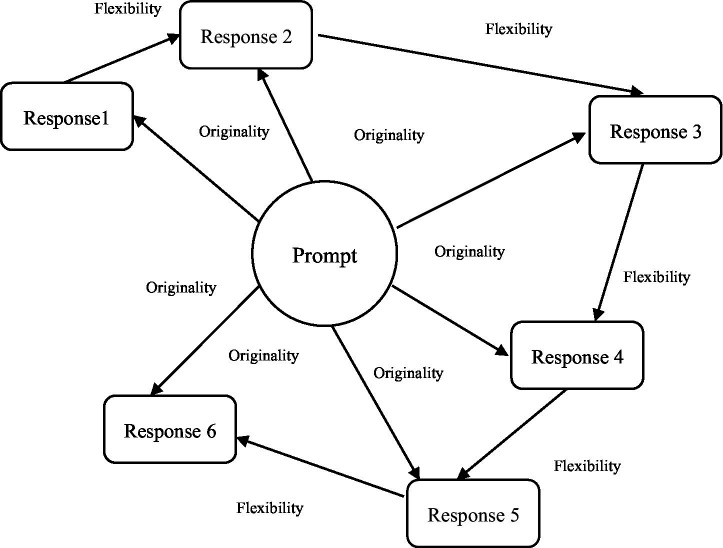
Flexibility as semantic distances between consecutive answers. The differing lengths of the arrows are meant to conceptually depict the varying semantic distance between prompt and responses for originality and between responses for flexibility. The model is based on Acar and Runco (2014).

In the current study, we created response pairs from the list of responses for each participant to assess flexibility of thinking. Similarly, to Johnson et al. (2021) who in their study examined metacognitive processes and operationalized idea diversity as the semantic distance between two responses, our first-word pair contained the first response and the second response. The second pair we created contained the second and third responses; the third pair contained the third and fourth responses, and so on (Figure 2). Next in our process of generating flexibility scores, semantic distance between the response pairs were calculated *via* a text-mining model, which becomes the flexibility score for those consecutive responses. We preserved the order in which the responses were given as creative idea generation is a process based on semantic search (Figure 2). This sequence of generated ideas holds important information about the participant’s ideation (Gray et al., 2019), indicating a temporal relationship among successive responses (Acar and Runco, 2014; Acar et al., 2019; Beaty et al., 2021; Olson et al., 2021).

To validate this scoring methodology, we used the GLoVe text-mining system to calculate semantic distance of adjacent responses (Pennington et al., 2014). GLoVe has been shown to mimic human raters in DT tasks and is currently considered superior to many other existing text-mining systems based on recent methodological investigations (Dumas et al., 2020b; Acar et al., 2021; Beaty and Johnson, 2021). We also plan to follow suggestions of Forthmann et al. (2019) to correct for multiword answers by stoplisting, which removes very commonly used words (e.g., the, and, as) from scoring. Term weighting was applied to enable incorporation of the frequency and importance of occurrences of each term. Averages of the weighted vectors were used to generate one vector representing multiword responses. As vector length does hold information about the detailedness of the response (Manning et al., 2008), some of this information could potentially be lost as a result of average weighting (Deerwester et al., 1990), however this methodology has been used successfully by many creativity researchers (Silvia et al., 2009; Dumas and Dunbar, 2014; Harbinson and Haarmann, 2014; Dumas and Strickland, 2018; Acar et al., 2021).

### Goals of current study

To examine the validity of our conceptualization and proposed measurement procedure for flexibility (OCS-Flex), obtained from the https://openscoring.du.edu/about site which is based on the GLoVe text-mining system, we conducted two different studies using DT tasks measuring creative ideation. In the first study, we focused on the convergent and discriminant validity of OCS-Flex. To do this, we correlated OCS-Flex with the established flexibility scoring method (Pub-Flex) of the verbal form of the Torrance Test of Creative Thinking (TTCT; Torrance, 1998), as well as TTCT fluency (Pub-Flu) and originality scores (Pub-Orig; the different scoring methods used in this study are listed in Table 1).

In the second study, DT was assessed with the AUT. These data were used to examine both the internal and external validity of OCS-Flex from a latent variable perspective. Following a factor analysis methodology, we calculated both composite and optimally-weighted reliability indices for the OCS-Flex model and correlated the resulting latent OCS-Flex scores to OCS-Flu, and OCS-Orig. Finally, we examined correlates of OCS-Flex to personality dimensions that have been previously shown to predict creativity (scoring methods used in the study are listed in Table 1).

Based on these aims the following research questions are posited and addressed:

1. What is the relation of the OCS-Flex to TTCT Pub-Orig and Pub-Flu scores and OCS-Orig and OCS-Flu score (discriminant validity)?
2. What is the relation of the OCS-Flex scoring to TTCT Pub-Flex scores (convergent validity)?
3. How well does the OCS-Flex scoring method fit into the theoretical framework of DT suggesting that items measure one latent construct (construct validity)?
4. How well can latent models capture the relationship between the three main dimensions of flexibility, originality, and fluency (construct validity)?
5. How well do scores from the OCS-Flex align with creative personality characteristics (criterion validity)?

## Study 1

### Methodology

The first study reanalyzed data from Acar et al. (2021). In the original study, two tasks of the Torrance Test of Creative Thinking-Verbal (TTCT), the Unusual Uses Test (UUT), and Just Suppose Test (JST), were scored for originality using three different text-mining systems. The authors compared the text-mining scores to snapshot and TTCT publisher-generated scoring. They found that all text-mining scores correlated strongly with snapshot and TTCT publisher-generated scoring, but the GLoVe text-mining system scores replicated established scoring methods most accurately. Acar et al. (2021) did not examine the flexibility of participants within this dataset, and the present analysis is therefore entirely novel. Scores generated for the current study are archived and can be accessed on Zenodo (LINK: DOI 10.5281/zenodo.6323425).

#### Participants

This dataset contained information from 224 participants; 101 of whom completed Form A and 123 of whom completed Form B of the TTCT Verbal (Torrance, 1998). Participants were undergraduate freshman students at a large public university in the Northeast United States. The average age of the sample was 18.20 (*SD* = 1.31) of which 26.7% of the participants were male, 48.9% female, and 24.4% did not report gender.

#### Measure and scoring

The TTCT Verbal (Torrance, 1998) contains six activities. In this study, data from only two of the activities, the JST and the UUT, were analyzed. The UUT solicits a list of differing uses for an everyday object. Participants had 5 min to list uses for Cardboard Boxes on Form A and Tin Cans on Form B. The JST presents a hypothetical situation and participants are prompted to list possible consequences of the event. On Form A, participants are asked what would happen if “.. the clouds had strings attached to them..” and on Form B if “..all we could see of people would be their feet..” Participants were given 5 min for each of the above activities to be completed. The answer booklets were scored by Scholastic Testing Service (STS), the publisher of the TTCT, for fluency (Pub-Flu), originality (Pub-Orig), flexibility (Pub-Flex) and follows the process described in the TTCT manual.^1^ STS raters are highly trained whose performance is measured against expert raters using an intraclass correlation of 0.90 or higher (Torrance, 1974). The reported scores were used in the study without any alteration. In addition, OCS-Flex scores were produced. To reflect the conventional summation scoring used in the TTCT publisher-generated scores, we calculated a sum score for both OCS-Flex and OCS-Orig. To overcome a potential fluency confound, mean scores were created for every participant by summing the OCS-Flex scores for each word pair and dividing it by OCS-Flu scores on each task separately (i.e., to indicate the average flexibility per item on the measure). Maximum OCS-Flex scores were also generated by choosing the individual response with the highest OCS-Flex score for each participant on each task. All responses were scored using the freely available web-based tool by Organisciak and Dumas (2020; https://openscoring.du.edu/about).

### Results

Results of the item-level descriptive statistics for each scoring methodology, listed in Table 1, are displayed in Table 2. In the first study, we correlated OCS-Flex (mean, maximum, and sum), OCS-Orig (mean, maximum, and sum), and Pub scores (flexibility, fluency, and originality). As some of the items violated normality, we used Spearman’s Rho.

**Table 2:** Item level descriptive statistics for studies 1 and 2.

		Flexibility			Originality			Fluency	
		Publisher-generated (Sum)	Mean	Max	Publisher-generated (Sum)	Mean	Max	Mean	Max
TTCT	UUT	8.46 (7.97)	0.21	0.86	9.32 (9.83)	0.75	0.84	11.16	34
	JST	8.61 (6.56)	0.23	0.80	8.34 (0.7.00)	0.74	0.91	9.82	27
AUT	Book	–	0.81	0.88	–	0.70	0.86	8.72	33
	Rope	–	0.67	0.86	–	0.64	0.83	8.33	21
	Fork	–	0.70	0.86	–	0.76	0.90	7.24	26
	Table	–	0.69	0.85	–	0.70	0.81	8.05	27
	Pants	–	0.66	0.85	–	0.64	0.91	7.05	23
	Bottle	–	0.73	0.93	–	0.69	0.86	7.46	28
	Brick	–	0.72	0.93	–	0.71	0.88	6.97	24
	Tire	–	0.67	0.83	–	0.70	0.85	6.70	26
	Shovel	–	0.67	0.83	–	0.74	0.89	6.26	34
	Shoe	–	0.71	0.86	–	0.69	0.92	6.89	27

TTCT, Torrance Test of Creative Thinking; UUT, Unusual Uses Test; JST, Just Suppose Test; AUT, Alternative Uses Test.

#### Relations among publisher-generated (pub) and text-mining flexibility (OCS-flex) scores

TTCT Pub-Flex scores correlated significantly with all OCS-Flex scores supporting reliability of the proposed scoring methods (Table 3). By far, the strongest correlation was observed between sum OCS-Flex scoring and Pub-Flex scores on both the UUT (*ρ* = 0.72, *p* < 0.01, 95% CI [0.63, 0.79]) and JST (*ρ* = 0.70, *p* < 0.01, 95% CI [0.60, 0.78]). As publisher scores are calculated by adding all scores together, the strong relationship between Pub scores and sum OCS-Flex scores is reasonably expected.

**Table 3:** Correlations of mean and maximum flexibility and originality scores with publisher-generated scores of the TTCT verbal.

	Text-mining mean scores		Text-mining maximum scores		Text-mining sum scores		Publisher-generated scores		
	Flexibility UUT(JST)	Originality UUT(JST)	Flexibility UUT(JST)	Originality UUT(JST)	Flexibility UUT(JST)	Originality UUT(JST)	Flexibility UUT(JST)	Originality UUT(JST)	Fluency UUT(JST)
*Text-mining mean scores*									
Flexibility UUT (JST)	1.000	0.054 (0.030)	0.555^**^ (0.673^**^)	0.470^**^ (0.381^**^)	0.435^**^ (0.383^**^)	0.225^*^ (0.113)	0.249^*^ (0.184^*^)	0.250^*^ (0.108)	0.205^*^ (0.185^*^)
Originality UUT (JST)		1.000	0.059 (0.049)	0.370^**^ (0.277^**^)	0.438^**^ (0.419^**^)	0.741^**^ (861^**^)	0.416^**^ (0.579^**^)	0.367^**^ (0.596^**^)	0.376^**^ (0.585^**^)
*Text-mining maximum scores*									
Flexibility UUT (JST)			1.000	0.302^**^ (0.395^**^)	0.481^**^ (0.517^**^)	0.115 (0.167)	0.289^**^ (0.233^*^)	0.292^**^ (0.274^**^)	0.278^**^ (0.233^*^)
Originality UUT (JST)				1.000	0.420^**^ (0.359^**^)	0.141 (0.227^*^)	0.483^**^ (0.390^**^)	0.412^**^ (0.417^**^)	0.395^**^ (0.390^**^)
*Text-mining sum scores*									
Flexibility UUT (JST)					1.000	0.152 (0.156)	0.720^**^ (0.702^**^)	0.828^**^ (0.731^**^)	0.813^**^ (0.709^**^)
Originality UUT (JST)						1.000	0.390^**^ (0.461^**^)	0.339^**^ (0.462^**^)	0.360^**^ (0.464^**^)
*Publisher-generated scores*									
Flexibility UUT (JST)							1.000	0.825^**^ (0.978^**^)	0.831^**^ (0.988^**^)
Originality UUT (JST)								1.000	0.979^**^ (0.980^**^)
Fluency UUT (JST)									1.000

UUT, Unusual Uses Test; JST, Just Suppose Test.

* *p* < 0.05 level.

** *p* < 0.01. (2-tailed).

Text-mining scores on the JST showed a consistently weaker although still significant correlation with publisher scores. The magnitude of differences in correlations between the UUT and the JST activities possibly depict the difficulty of scoring the more elaborate and complex JST. These findings are also in line with the study by Landreneau and Halpin (1978) reporting that the average flexibility, fluency, and originality scores on the JST were consistently lower on the TTCT compared to the UUT. Silvia (2011) also found that UUT achieved the highest reliability while JST had the poorest reliability using latent modeling techniques. Finally, in the study by Acar et al. (2021), the inter-rater reliability of the JST using the TTCT was lower compared to the UUT.

#### Relations among flexibility, originality, fluency scores

##### Publisher-generated (Pub) scores

Correlations of Pub-Orig, Pub-Flu, and Pub-Flex revealed very strong relations among the dimensions which ranged from 0.83 to 0.99. Activity-specific correlations between the TTCT Pub scores (flexibility, fluency, and originality) ranged between 0.83 and 0.98 on the UUT and between 0.98 and 0.99 on the JST (Table 3).

##### Text-mining (OCS) scores

The correlation between OCS-Orig and OCS-Flex for mean and the sum scoring was not significant on both tasks (UUT and JST; Table 3). However, when using maximum scoring the relation was significant although weak for both the UUT (*ρ* = 0.30, *p* < 0.01, 95% CI [0.18, 0.41]) and JST (*ρ* = 0.40, *p* < 0.01, 95% CI [0.28, 0.50]). Fisher’s transformation revealed that this correlation was significantly smaller than correlations between Pub-Flex and Pun-Orig scores for both UUT (*z* = 9.05, *p* < 0.05) and JST (*z* = 19.25, *p* < 0.05).

The sum OCS-Flex scores correlated the strongest with OCS-Flu scores for both the UUT (*ρ* = 0.81, *p* < 0.01, 95% CI [0.76, 0.85]) and JST (*ρ* = 0.71, *p* < 0.01, 95% CI [0.64, 0.77]). This scoring, similarly, Pub scores, is calculated by adding all flexibility scores together and therefore provides no correction for the confounding effects of fluency. Mean and maximum OCS-Flex scores correlated weaker although significantly with OCS-Flu (Table 3). Fisher’s transformation revealed that both maximum (*zuut* = 9.52, *p* < 0.05; *zjst* = 10.23, *p* < 0.05) and mean scoring (*z_uut_* = 10.34, *p* < 0.05; *z_jst_* = 10.56, *p* < 0.05) correlated significantly weaker with OCS-Flu than Pub-Flex scores. These results suggest that both mean and maximum OCS-Flex scoring provided differentiation from both OCS-Orig and OCS-Flu, therefore, outperforming Pub scores.

## Study 2

### Methodology

For the second study, we re-analyzed data collected by Dumas et al. (2020a). The data is archived and can be accessed on Zenodo (LINK: https://zenodo.org/record/3899579#.X3M81KSlaQ; DOI:10.5281/zenodo.3899578). Dumas et al. (2020a) used text-mining to examine originality on the AUT. The study compared originality of three different groups of participants (professional actors, student actors and non-actor controls), however they did not consider the flexibility of these participants, and therefore the present analysis is entirely novel.

#### Participants

In the second study, we used a dataset that included information from 296 participants across three groups: non-acting adults (*n* = 92), undergraduate acting and theater major students (*n* = 100), and professional actors (*n* = 104). Sixty-three percent of the sample were female, with an average age of 28 (SD = 6.40). Eighty-six percent of the participants were white, 6% African American, 5% Latinx, and 5% Asian. All individuals were financially compensated for their participation.

#### Measures and scoring

The Big Five Aspects Scale (DeYoung et al., 2007) contains 100 items and measures characteristics mapped onto the Big Five personality traits including: Neuroticism, Agreeableness, Conscientiousness, Extraversion, and Openness (Goldberg, 1990). These aspects are further subdivided into two facets each: the Neuroticism attribute contains Volatility and Withdrawal; the Agreeableness trait comprises Compassion and Politeness; the Conscientiousness characteristic measures Industriousness and Orderliness; the Extraversion attribute contains Enthusiasm and Assertiveness; and the Openness aspect is divided into Intellect and Openness. The 10 facets displayed strong internal consistency reliability (ranging between 0.85 to 0.95; Dumas et al., 2020a,b).

The AUT is one of the oldest and most widely used assessments in creativity research (Guilford, 1967; Silvia et al., 2008). This task is very similar to the UUT as it asks participants to generate different uses for everyday object. In this version of the AUT, participants were asked to generate as many original or unusual uses as they could to 10 everyday objects: book, fork, table, hammer, pants, bottle, brick, tire, shovel, and shoe. The time limit on this measure was 2 min per object. The measure was scored along four dimensions: fluency, originality, and flexibility.

To score the items in this study, the same freeware system was used as in Study 1 (Organisciak and Dumas, 2020; https://openscoring.du.edu/about). OCS-Flu was assessed as the count or number of responses each participant provided to each of the items. Mean OCS-Flu was measured as the average fluency across all tasks. The average was calculated as the sum of all OCS-Flu scores across all 10 items divided by the number of items (10). Maximum OCS-Flu indicates the highest fluency score achieved among the 10 tasks. OCS-Orig was calculated as the semantic distance between the prompt and each response (Dumas et al., 2020a,b). For mean OCS-Orig, we summed the scores and divided each by fluency on each task. Maximum OCS-Orig denotes the highest scores on each of the 10 tasks. Internal consistency reliability for OCS-Orig mean and OCS-Orig maximum scores were 0.85 and 0.86, respectively (Dumas et al., 2020a,b). Similar to the first study, word pairs were created to measure semantic distance and mean, and maximum flexibility scores were generated for each of the 10 AUT items.

### Results

Item-level descriptive statistics for each measurement methodology employed in the study (Table 1) are displayed in Table 2. Many of the items displayed higher than acceptable skewness on both mean OCS-Flex and maximum OCS-Flex scoring. To account for the non-normal distribution, Spearman’s Rho correlations were used.

#### Reliability and validity of text-mining flexibility (OCS-flex) scores

Reliability of the AUT across 10 items was evaluated using Cronbach’s alpha (Cronbach, 1951), Hancock’s H (Hancock and Mueller, 2001), and omega of McDonald (1999). Taken together, these three indices encompass the theoretical range of the score reliability. Both OCS-Flex scoring methods achieved good reliability on all reliability indices (Table 4).

**Table 4:** Model fit statistics.

Model		Reliability			*x^2^*			RMSEA		CFI	TLI	SRMR
		*α*	*H*	*ω*	Value	*df*	*p*	Value	95%CI			
Mean flexibility		0.823	0.845	0.828	45.189	35	0.116	0.083	[0.000, 0.055]	0.960	0.948	0.054
Max flexibility		0.799	0.822	0.808	48.060	35	0.070	0.036	[0.000, 0.058]	0.958	0.946	0.045
Mean model	Flexibility	0.823	0.841	0.827	620.87	372	<0.05	0.058	[0.041, 0.054]	0.940	0.930	0.070
	Originality	0.843	0.887	0.856								
	Fluency	0.952	0.955	0.952								
Max model	Flexibility	0.749	0.789	0.759	553.67	372	<0.05	0.041	[0.033, 0.048]	0.946	0.937	0.043
	Originality	0.832	0.876	0.842								
	Fluency	0.952	0.955	0.952								

*α*, Cronbach’s Alpha; H, Coefficient H; *ω*, Coefficient Omega; RMSEA, root-mean-square error of approximation; CFI, comparative fit index; TLI, Ticker Lewis index; SRMR, standardize root-mean-square residual; Mean Model, modeling including mean flexibility, mean originality and fluency; Max Model, modeling including maximum flexibility, maximum originality and fluency.

**Figure 3:**
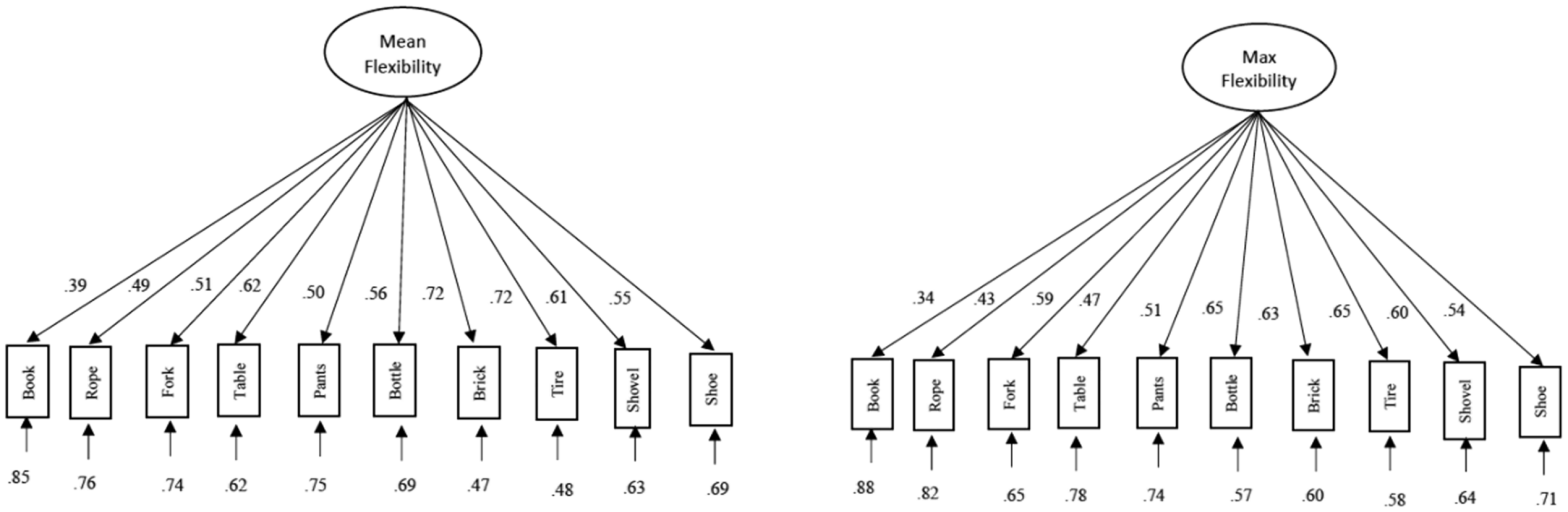
Path diagram of mean and maximum flexibility scoring.

Construct validity of these 10 AUT items was assessed *via* confirmatory factor analysis (CFA) with Mplus 8.4 software (Muthén and Muthén, 2007). As normality was violated for many of the AUT items, robust maximum likelihood estimations with Santorra-Bentler corrections were used to model the data. To evaluate our OCS-Flex mean and maximum models (Figure 3), we used conventional cutoff values to assess model fit (Hu and Bentler, 1999). Although these fixed cutoffs for fit indices have been widely used, their generalizability is limited as values are based on one specific confirmatory factor analysis model therefore, results should be interpreted in conjunction with reliability indices and factor loadings. Although, specific model cutoffs can be calculated using the Shiny app by McNeish and Wolf (2020) it is only accurate for models estimated using maximum likelihood.

Chi-square goodness of fit statistics, for both methods, were not significant although root mean square error of approximation (RMSEA) was below the conventional cut off of 0.06 while the comparative fit index (CFI) and the Tucker-Lewis index (TLI) were above the conventional suggested cut-off value of 0.90 and the standardized root mean square residual (SRMR) were below the conventional cut-off of 0.08 (Table 4). Although all indices suggested a good fit for both of our models, not all items load equally well onto the OCS-Flex factor. The item “book” had to lowest loading for both mean (0.39) and maximum (0.34) onto OCS-Flex suggesting that this item might not measure flexibility well.

#### Relations among text-mining flexibility (OCS-flex), originality (OCS-Orig), fluency (OCS-flu) scores

**Figure 4:**
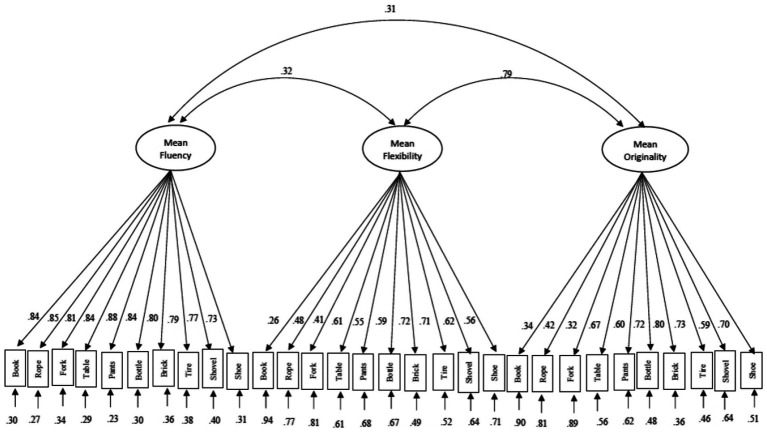
Path diagram of mean flexibility, mean originality, and fluency scoring.

To further examine relations among the three most often used dimensions of DT (fluency, originality, and flexibility) we constructed CFA models with OCS-Flex, OCS-Orig, and OCS-Flu correlated latent factors. A model was fitted with scores for mean OCS-Flex, mean OCS-Orig, and OCS-Flu and another for maximum OCS-Flex, maximum OCS-Orig, and OCS-Flu. The three correlated factors each contained 10 items (Figures 3, 4). It is reasonable to assume that the 10 items on the AUT share variance across the factors. Therefore, each item was correlated with the same item loading on the other two factors. No cross-loading or correlations between items on the same factor was allowed as variances across items should be accounted for by the factor onto which they load. These strong a-prior specifications resulted in models with good reliability (Cronbach’s alpha, Hancock’s H, and McDonald’s Omega) with the exception of the acceptable fit for the OCS-Flex dimension in the maximum model (Table 4). Chi-square goodness of fit statistics was significant for both models while RMSEA, comparative fit index (CFI) and the Tucker-Lewis index (TLI), SRMR all displayed a good fit for both methods (Hu and Bentler, 1999; Table 4).

The correlation between the OCS-Flex and OCS-Orig factors in the mean model (0.79) and between OCS-Flex and OCS-Flu factors in the maximum model (0.63) was both strong suggesting a persistent relationship between these factors. Although items loaded on the OCS-Flu factor well, item loading varied across the OCS-Flex and OCS-Origi factors for both models. Similar to the single factor model, the item “book” loaded the weakest on both OCS-Flex and OCS-Orig on both mean and maximum models (Figures 3, 4).

#### Criterion validity

To further expand on the examination of criterion validity, scores of mean OCS-Flex and maximum OCS-Flex were correlated with 10 facets of personality associated with the Big Five model (i.e., openness, intellect, enthusiasm, assertiveness, industriousness, orderliness, compassion, politeness, volatility and withdrawal; DeYoung et al., 2016). Mean OCS-Flex significantly positively correlated with openness (*r*(296) = 0.15, *p* = 0.047, 95% CI [0.04, 0.26]) while maximum OCS-Flex scores positively correlated with the enthusiasm facet of the extraversion factor (*r*(296) = 0.17, *p* = 0.003, 95% CI [0.06, 0.28]).

## Discussion

In this study, we proposed and psychometrically validated an automated text-mining based approach to score flexibility on DT tasks. Based on our evaluation of these methods, the following key findings have emerged.

### Text-mining based flexibility (OCS-flex) scores closely correlate with TTCT Publisher scoring (pub)

Due to its extensively studied validity, the TTCT publisher-generated scores were used to validate OCS-Flex scores in our first Study (Chase, 1985; Treffinger, 1985; Torrance, 1998; Cramond et al., 2005; Kim, 2006; Acar et al., 2022). All proposed OCS-Flex scores (mean, maximum, sum) correlated significantly with Pub-Flex scores providing proof of the validity of our scoring method. Publisher-generated scores are calculated as the sum across games of the TTC verbal, therefore, it was not surprising that our sum OCS-Flex score correlated the strongest with publisher scores (Table 3).

There were very high inter-correlations among the TTCT Pub-Flex, Pub-Flu, and Pub-Orig scores ranging between 0.83 and 0.99. The strong correlations among the DT dimensions are not unique to our current study and the lack of differentiation among dimensions on the TTCT has been long written about in the field (Mansfield et al., 1978; Clapham, 1998; Mouchiroud and Lubart, 2001; Kim, 2006; Silvia et al., 2008; Zeng et al., 2011; Grajzel et al., 2022). Due to this strong correlation between Pub-Orig and Pub-Flex scoring, we were not able to establish discriminant validity of our OCS-Flex scoring method. However, for both the sum and mean text-mining scoring, the correlation between OCS-Orig and OCS-Flex was not significant suggesting that these scoring methods allow for better differentiation between dimensions. Even in the case of maximum scoring, the correlation between OCS-Flex and OCS-Orig was significantly smaller than the correlation between Pub scoring of the same dimensions.

The correlation of OCS-Flex and OCS-Orig with OCS-Flu was the strongest for sum scores. This is of no surprise as this scoring method does not provide any correction for fluency confound. The reduced correlations between OCS-Flu scores and mean and maximum scoring suggest that at least some fluency control has been implemented using these scoring methods. These results suggest that all OCS scoring methods proposed in Study 1 (mean, maximum, and sum) allow for differentiation among the three dimensions of originality, fluency, and flexibility.

### Text-mining based flexibility (OCS) scores demonstrated high reliability and construct validity

**Figure 5:**
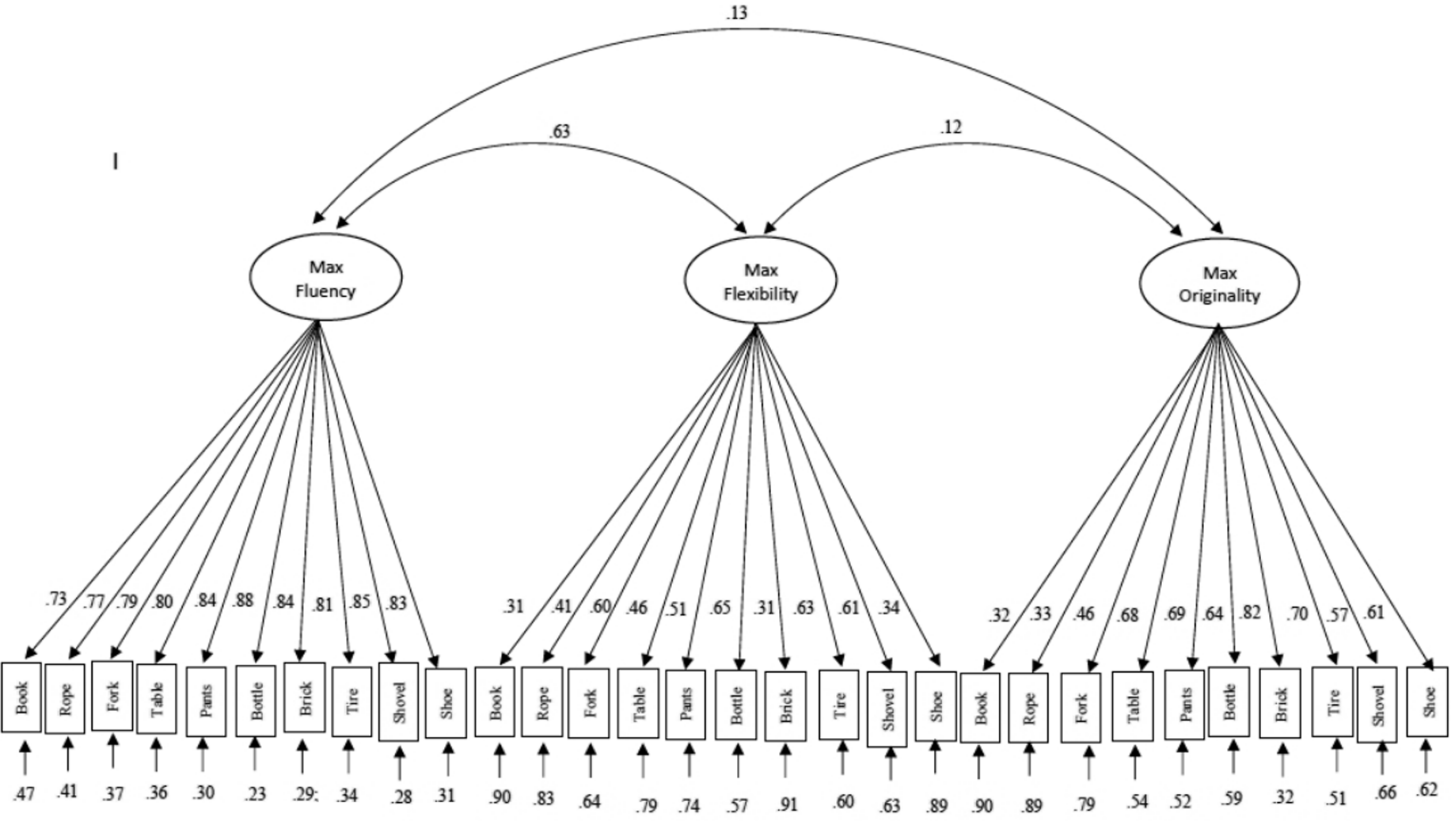
Path diagram of maximum flexibility, maximum originality, and fluency scoring.

To evaluate construct reliability and construct validity of OCS-Flex, two CFA models were constructed. The first model included all mean OCS scores for all 10 items of the AUT while the second model contained all maximum OCS scores. Both models displayed a good fit to the data as indicated by model fit indices, internal reliability indices (Cronbach’s alpha, coefficient H and Omega; Table 4), and factor loadings (displayed in Figure 5). However, some of the item loadings were lower (e.g., book), suggesting that not all items measure the concept of flexibility equally well and highlighting the need for more latent measurement modeling in this area of research.

### Text-mining based flexibility (OCS) scores showed valid relations with external variables

To examine the relationship between OCS-Flex, OCS-Orig, and OCS-Flu scores two further latent models were constructed (Figures 3, 4) These models, one including mean OCS-Flex, mean OCS-Orig, and OCS-Flu and the other containing maximum OCS-Flex, maximum OCS-Orig, and OCS-Flu displayed good to acceptable fit based on reliability indices (Cronbach’s alpha, coefficient H and Omega), fit indices and factor loadings (Table 4). There was a strong correlation between OCS-Flex mean and OCS-Orig mean latent factors. This strong relationship between originality and flexibility has been observed previously and could be accounted for by the theoretical relationship between the two dimensions (Runco, 1986b; Acar and Runco, 2014; Acar et al., 2022). The correlation observed between maximum OCS-Flex and OCS-Flu factors suggests that some of the fluency confound still remained using this scoring method.

To further expand on the examination of criterion validity, mean OCS-Flex and maximum OCS-Flex scores were correlated with personality characteristics of openness, intellect, enthusiasm, assertiveness, industriousness, orderliness, compassion, politeness, volatility, and withdrawal. Mean OCS-Flex was significantly positively correlated with openness to experiences. Openness has been long thought of as one of the main personality characteristics of creative individuals suggesting that open-minded and curious participants generate more creative responses (McCrae, 1987; King et al., 1996; Dollinger et al., 2004; Hagtvedt et al., 2019). Maximum OCS-Flex scores positively correlated with enthusiasm. Grohman et al. (2017) found students’ creative behavior was correlated with teacher reported enthusiasm. Passion or enthusiasm is also closely related to intrinsic motivation (Amabile and Fisher, 2000; Moeller et al., 2015) which has been implicated in creative idea generation (Paulus and Brown, 2007; Dumas et al., 2020a; Gu et al., 2020).

### Limitations and future directions

Despite the strong results supporting the two OCS-Flex scoring methods (mean and maximum) presented in this study, the specific foci of the current work necessarily imply that many questions regarding flexibility scoring are still awaiting examination. Although the psychometric findings we reported here suggest that these scores are ready to be deployed in research, we also put forth a number of future directions in the measurement of flexibility that may be worth considering.

#### Targeted focus on verbal measures

Verbal DT tasks are the most often utilized measures assessing creative potential (Hass, 2017; Reiter-Palmon et al., 2019; Acar et al., 2020). Therefore, we focused our attention on verbal tasks only, examining data gathered with two of the most popular assessments (TTCT and researcher-created AUTs; Mouchiroud and Lubart, 2001; Cho et al., 2010; Cortes et al., 2019). Of course, the application of text-mining methods here also necessitates the use of verbal or written data, because the process fundamentally relies on the words participants use to respond to the task. So, it remains to be explored how figural DT scores might be related to text-mining verbal scores and specifically to flexibility using the mean and maximum scoring method explored in this article (Cropley and Marrone, 2022).

#### Modeling time between responses

There has been an emerging interest in recent years exploring the relation between time spent on tasks and flexibility of responses. Beketayev and Runco (2016) found a positive correlation between latency (i.e., the time between responses to a DT task) and flexibility which diminished after 178 s. Acar and Runco (2017) found that category switching was denoted by an approximate increase of 5 s spent on a given response. Said-Metwaly et al. (2020) conducted a meta-analytic study and found that longer time allowed on task increased flexibility of responses. Data used in this study did not allow for the examination of the length of time per idea generated. Therefore, future research should focus on exploring the relationship between latency and text-mining based flexibility scores.

#### Semantic vs. categorical flexibility

Vector based semantic methods, such as the one used in this study, allow for quantitative expressions of semantic distance therefore providing an automatic and valid way to measure DT (Forster and Dunbar, 2009; Beketayev and Runco, 2016; Forthmann et al., 2019; Dumas et al., 2020b; Acar et al., 2021). Text-mining methods however also present certain restrictions to measuring flexibility. The vector-based method we used here was only able to model semantic distances between two adjacent ideas (and then average those distances across all idea pairs), and therefore categories or clusters of ideas that could be sorted into conceptual groups were not detected (Kenett, 2019). For this reason, we propose that flexibility could be examined from multiple perspectives by combining LSA-based text-mining methods and associative-based semantic network models (Kenett, 2019; Kumar et al., 2020). This is especially important as although LSA-based text-mining methods have been successfully used in many DT studies, others have cautioned of their limited ability to capture the wide complexity of the semantic systems (Hutchison et al., 2008). Moreover, by not accounting for the clustering of the responses in our current methodology, flexibility can be overestimated. Therefore, future research should also examine the bias possibly embedded in this proposed scoring method due to the use of consecutive pairs of responses to calculate flexibility.

## Conclusion

Flexible thinking patterns are a hallmark of creative cognition (Benedek et al., 2014; Chrysikou, 2018; Gube and Lajoie, 2020; Huang et al., 2020). Flexibility however is less often assessed in DT tasks compared to other dimensions such as fluency and originality (Reiter-Palmon et al., 2019). Flexibility provides a unique and important measure that can differentiate the creative quality above and beyond originality related to the metacognitive process of switching (Nijstad et al., 2010; Kenett et al., 2018; Forthmann et al., 2019).

In this study, we proposed an automated scoring for flexibility which provides an alternative, reliable, valid, and practical way to measure flexibility on DT tasks. We provided evidence that our scoring method aligns with existing standards employed by researchers for scoring flexibility (i.e., number of categories on TTCT responses) and that this method is able to mimic relationships between dimensions (fluency, originality) of DT. Latent structure of this flexibility scoring followed theoretically agreed-upon dimensionality and captured real-life differences in personality among participants. This methodology enables the more extensive use of this dimension and provides a less labor intensive and costly measurement of flexibility. Our proposed scoring methodology does not suffer from the sample dependence issues of uniqueness scoring or from rater subjectivity in subjective scoring, therefore, proving a scoring methodology that is comparable across studies and easy to implement. Given the psychometric evidence presented here and the practicality of automated scores, we recommend adopting this new method. Surely, further investigations are needed to examine additional evidence of validity.

## Data availability statement

The original contributions presented in the study are included in the article/supplementary material, further inquiries can be directed to the corresponding author.

## Author contributions

All authors listed have made a substantial, direct, and intellectual contribution to the work, and approved it for publication.

## Conflict of interest

The authors declare that the research was conducted in the absence of any commercial or financial relationships that could be construed as a potential conflict of interest.

## Publisher’s note

All claims expressed in this article are solely those of the authors and do not necessarily represent those of their affiliated organizations, or those of the publisher, the editors and the reviewers. Any product that may be evaluated in this article, or claim that may be made by its manufacturer, is not guaranteed or endorsed by the publisher.

## Footnotes

^1^ https://ststesting.com/reportssmp.html#TTCT
